# Building capacity for local public health: lessons from a mixed-methods evaluation of an academic-public health partnership used in response to COVID-19

**DOI:** 10.3389/fpubh.2024.1387371

**Published:** 2024-07-31

**Authors:** Sarah Fielman, Patricia A. Elliott, Alyson Codner, Hiba Abousleiman, Ally Cogan, Zoë Wangstrom, Jacey A. Greece

**Affiliations:** Department of Community Health Sciences, School of Public Health, Boston University School of Public Health, Boston, MA, United States

**Keywords:** COVID-19, local public health, academic-practice partnerships, Consolidated Framework for Implementation Research (CFIR), mixed-methods program evaluation

## Abstract

**Introduction:**

The Massachusetts Academic Health Department Consortium (AHD) established the Academic Public Health Volunteer Corps (APHVC) to support Local Health Departments (LHDs, *n* = 351) to meet rapidly emerging needs during the COVID-19 pandemic through engaging student volunteers. A program evaluation captured lessons learned and informed recommendations for sustainability and future replication.

**Methods:**

The mixed-methods evaluation leveraged the Consolidated Framework for Implementation Research (CFIR). Data were stratified by LHD engagement with APHVC. Quantitative surveys informed probes for qualitative focus groups and interviews; findings were categorized into CFIR constructs using a deductive approach.

**Results:**

One-fifth of LHDs (*n* = 76, 27 used APHVC services, 45 did not) completed the survey. Eleven employees participated in follow-up focus groups or interviews. APHVC filled resource gaps, built capacity, and provided high-quality deliverables. LHDs experienced issues with reliability and communication of volunteers and lacked time to train volunteers.

**Conclusions:**

CFIR aided in evaluating APHVC in real time, producing actionable recommendations for best practices, dissemination, and future iterations of the program. Results are being used to enhance program effectiveness and sustainability, community health, and health equity across Massachusetts, and may help inform academic practice-based programs across the United States.

## Introduction

Local health departments (LHDs) are governmental entities that play a central role in supporting the health of communities and address health areas ranging from infectious diseases to injury prevention to healthcare access (1, 2). There are over 2,800 LHDs (2) in the United States; they are consistently underfunded and understaffed (2, 3), hindering timely responses during public health emergencies like the COVID-19 pandemic.

LHD challenges were exacerbated during the pandemic, with 76% of LHDs reporting insufficient staffing and 47% reporting a lack of dedicated pandemic funding (4). They had inconsistent and inadequate guidelines and communication from federal and state governments (4, 5); a need for increased staff capacity in resource management, diversity and inclusion, and effective communication (6); and, a lack of data and community partnerships that added additional burdens (4). About 70% of LHDs expanded the provision of emergency preparedness and routine epidemiologic and surveillance services to respond to the pandemic (4, 7). This interfered with the provision of other key LHD services such as obesity prevention (75% of LHDs reduced services), high blood pressure screening (67%), blood lead screening (61%), environmental health inspections (48%), and immunizations (47%) (4, 7).

Massachusetts has 351 LHDs that are responsible for unique jurisdictions within the state, the highest number in the United States (4, 7). Due to this decentralized system, Massachusetts LHDs faced obstacles and inefficiencies in sharing resources and practices, despite reporting increased communication during the pandemic (8). LHDs reported that a centralized, regional approach to COVID-19 would have resulted in more streamlined efforts and resource-sharing, uniformity in messaging and policy implementation, and improved coordination and standardization of public health training (7).

Historically, academic-practice partnerships have proven beneficial in a variety of under-resourced contexts. For example, clinically-oriented partnerships pair nursing students and medical trainees with local hospitals (8–12), and non-clinical partnerships pair public health students or university researchers with LHDs (10, 13, 14). These alliances demonstrate benefits for all stakeholders; students and early career professionals gain valuable hands-on skills and infuse the workforce with innovative ideas, while organizations gain personnel, new perspectives, and support for infrastructure (15).

In response to pandemic-related challenges and building on the known benefits of practice-academic linkages, the Academic Health Department Consortium (AHD), which is comprised of the Massachusetts Department of Public Health and thirteen academic institutions, launched the Academic Public Health Volunteer Corps (APHVC) in March 2020 (16). APHVC paired public health students and alumni volunteers with LHDs seeking additional resources (17). This novel partnership was designed to benefit LHDs and early public health professionals alike (16).

APHVC underwent a mixed-methods evaluation (MME) guided by the Consolidated Framework for Implementation Research (CFIR) (18) to identify successes and areas for growth from the perspectives of Massachusetts LHDs that did and did not utilize APHVC. The program evaluation aims were to identify the diverse needs of LHDs during the COVID-19 pandemic and the extent to which APHVC fulfilled those needs; identify barriers to uptake of APHVC and future motivations for engagement among those who used and did not use APHVC; and, issue recommendations regarding sustainability, improvement, and replication to the APHVC implementing agencies. Evaluation findings ensure that future iterations of APHVC support LHDs in accessing resources and sustaining services.

## Methods

### Program description

In March 2020, the AHD established APHVC in response to the COVID-19 pandemic (16). The academic-practice based partnership aimed to leverage a large volunteer base of students and alumni to assist, enhance, and expand local health efforts (19). While APHVC initially focused on COVID-19 testing and community contact tracing (7), it quickly expanded to include additional response efforts, such as community outreach, message development, data collection, and analysis. For APHVC volunteers, the program's goal was to provide hands-on experience and exposure to LHD work, creating opportunities for career development, ultimately strengthening the public health workforce pipeline.

### Conceptual framework

To understand the challenges and successes of APHVC, a mixed-methods evaluation (MME) was conducted according to the Consolidated Framework for Implementation Research (CFIR), which assists in providing insights into specific factors that influence program implementation of an intervention to encourage future effective enhancements (18). It is regularly used to systematically analyze and organize program implementation findings, particularly those with a large environmental influence.

This program evaluation was conducted before the current CFIR updates (20), and the established CFIR (18) was used to understand the perspectives of LHDs on APHVC, identify the factors that influenced APHVC program use or non-use, and present cohesive and actionable recommendations for future program iterations. This was accomplished through consideration of the five CFIR domains: characteristics of the intervention, outer/inner setting, implementation climate, characteristics of individuals, and process. Across those domains, there are nearly 40 constructs that represent different components of influence for implementation of a program like APHVC (18). Therefore, a *menu of constructs* approach was used (21) in which pre-determined constructs are used for the development of surveys and interview guides to assist in focusing data collection and analysis on the most relevant constructs.

### Study design

The MME used quantitative and qualitative assessments to explore contexts and perspectives of LHDs. The authors collaborated with implementing agencies in the design, development, conduct, and execution of the evaluation. The quantitative results provided probes for the qualitative assessments. In addition to using CFIR to guide data collection and analysis, a logic model that was developed collaboratively by the implementing agencies and the evaluation team, further synthesized the practice-focused outcomes and guided recommendations for future application. The program evaluation received exempt approval from human subjects research review by the Boston University Medical Campus Institutional Review Board.

### Study sample

The implementing agencies emailed a Qualtrics survey to contacts in all 351 Massachusetts LHDs, regardless of their engagement with APHVC. This allowed key LHD contacts to receive the evaluation invitation from a known and trusted sender. LHDs were given approximately three weeks to complete the survey with two reminders. Surveys were initiated by 118 respondents and completed by 72 LHDs (use = 27, non-use = 45).

Survey respondents who expressed interest in participating in a follow-up focus group or interviews were invited via email by the evaluation team to participate. Interviews were offered to those who could not join a focus group due to scheduling conflicts. Respondents were told responses were confidential. Participation was not incentivized. Focus groups and interviews were conducted over Zoom with two evaluation team members present. They facilitated discussions and transcribed the responses.

Respondents were categorized into use (worked for an LHD that used APHVC between March 2020 to August 2021) and non-use (worked for an LHD that did not used APHVC between March 2020 to August 2021) groups. Of the 72 LHD representatives that responded to the survey (use = 27, non-use = 45), 11 participated in focus groups and interviews (use = 5, non-use = 6).

### Data collection

The survey took < 15 min to complete and assessed the characteristics of LHDs and communities served, the impact of COVID-19 on LHD efforts and resources, experiences working with APHVC volunteers (when applicable), and the benefits and challenges of APHVC utilization (when applicable). Survey questions, organized by the five CFIR domains, were developed using pre-existing publicly-available surveys (22) and aligned with the program outcomes. Certain questions were answered by all respondents; a subset of questions was completed based on APHVC use.

Subsequent qualitative data collection extracted findings from survey respondents and provided contexts on the needs and perspectives of LHDs. A focus group guide was developed using data from the quantitative surveys, aligning questions with the five CFIR domains and using quantitative results to generate probes. Each session lasted between 30–90 min, and focused on gaps in public health infrastructure before and during COVID-19, reasons for engaging or not engaging with APHVC, challenges working with APHVC students, and resources and supports that should be considered for future engagement with the APHVC.

### Data analysis

Survey results were analyzed in SAS (version 9.2). Descriptive statistics were generated. For continuous data, means were calculated and chi-square tests and *t*-tests were performed to test for statistical significance (threshold set at *p* < 0.5). For categorical data, frequencies were used.

Stakeholder interviews were coded deductively, simultaneously line-by-line by two members of the evaluation team using a priori set of CFIR constructs (codes). Data were crossed referenced with CFIR domains and assessed for inclusion in the report of constructs, results, and recommendations. Coders determined if each code had a positive, negative, or neutral association with the implementation of APHVC. Discrepancies in categorization were discussed and resolved with a third member until consensus. After coding was complete, data were organized into analytic matrices for review and identifying implementation patterns, themes, and exemplar quotes (23).

## Results

### Survey findings

A total of 76 individuals completed the survey: 27 who used APHVC, 45 who did not, and 4 who were not sure.

**Figure 1:**
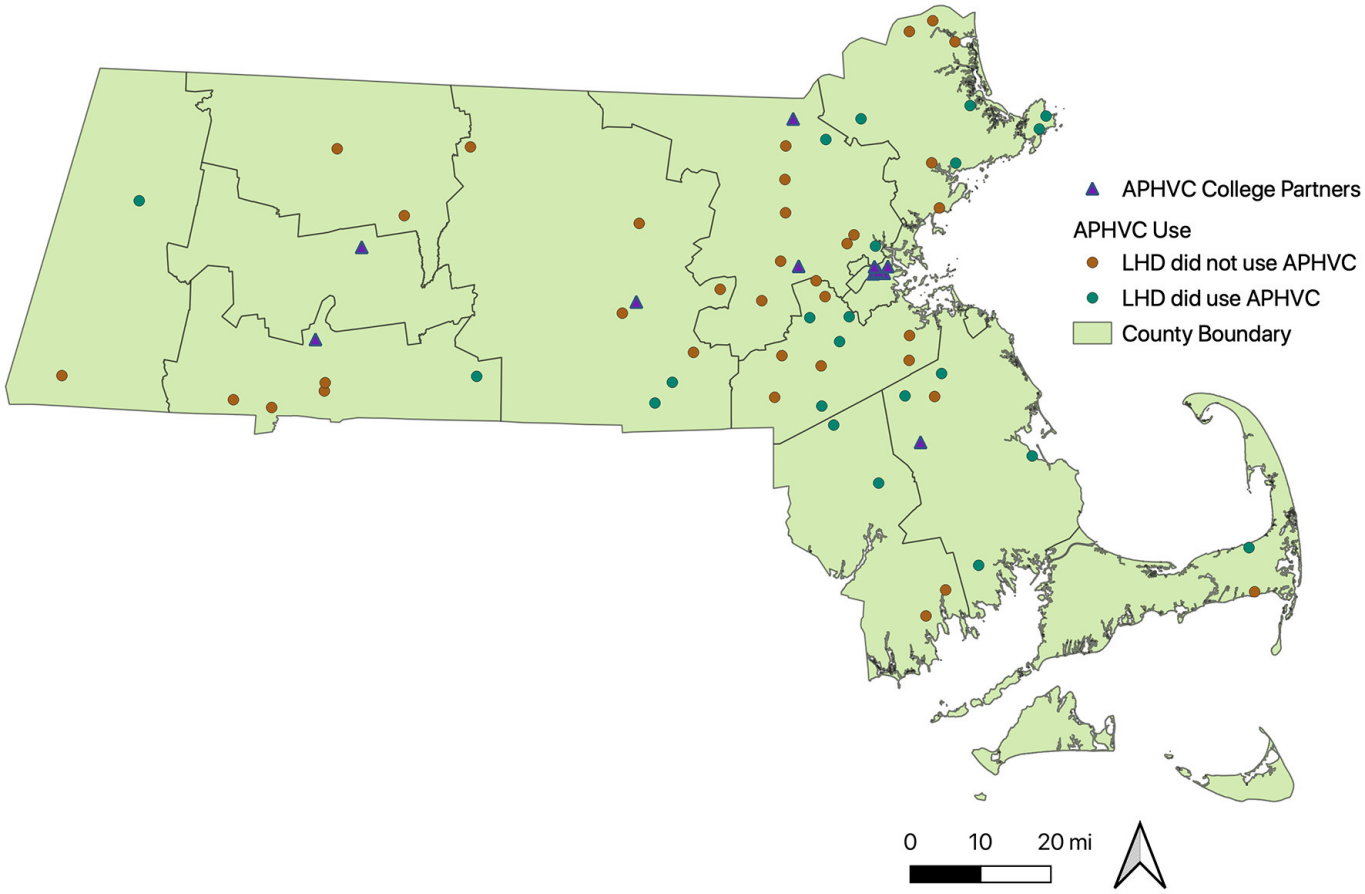
Locations of Academic Public Health Volunteer Corps Usage by Local Health Department (*n* = 351) and College Partners in Massachusetts between 2020–2021. Data sources: APHVC use—Survey data: Alyson Codner. College partnerships: https://academicpublichealthvolunteercorps.org/about/. Country Boundaries: https://www.mass.gov/info-details/massgis-data-counties#downloads-.

Survey respondents had a variety of job functions as defined by the Department of Public Health (directors 37%, nurses 22%, health agents 17%, board of health members 12%, registered sanitarian 12%, health officers 8%, consultants 3%, and other 11%); years of service at that LHDs (36% >10 years, 25.3% 1–3 years, and 17.3% 6–10 years); and, number of staff employed at an LHD varied by community (mean = 6 individuals; std = 10.65, min = 0 max = 60; Table 1). Most survey respondents (60%) did not use APHVC. Those who did and those who did not were broadly distributed across the state, with higher reports of use in Eastern Massachusetts (Figure 1). Location of academic partners did not appear to influence the use of APHVC.

**Table 1:** Characteristics of Local Health Department Respondents across Massachusetts from 2020 to 2021.

	**Total (*****N*** = **76)**		**Used APHVC (*****N*** = **27)**		**Did not use APHVC (*****N*** = **45)**	
	***N*** **(mean)**	**% (Std)**	***N*** **(mean)**	**% (Std)**	***N*** **(mean)**	**% (Std)**
**Job title or Role in Local Public Health**						
Health Officer	6	8.00	3	11.11	2	4.55
Health Agent	13	17.33	6	22.22	7	15.91
Registered Sanitarian	9	12.00	4	14.81	5	11.36
Nurse	17	22.67	3	11.11	12	27.27
Director	28	37.33	12	44.44	16	36.36
Consultant	2	2.67	0	0	2	4.55
Board of Health Member	9	12.00	4	14.81	4	9.09
Other	8	10.67	3	11.11	3	6.82
**Number of years working at the local or regional health department**						
< 1 year	7	9.33	2	7.41	4	9.09
1–3 years	19	25.33	7	25.93	9	20.45
3–5 years	9	12.00	3	11.11	6	13.64
6–10 years	13	17.33	5	18.92	8	18.18
More than 10 years	27	36.00	10	27.04	17	38.64
**Number of FT employees that work in your HD**	6.4	10.65	8.4	14.09	5.3	8.32
**HD regularly work with interns (excluding APHVC)**						
No	37	50.00	12	46.15	21	27.73
Yes	36	48.65	15	53.85	22	50.00
Don't know	1	1.35	0	0	1	2.27

Among respondents who used APHVC, 71% reported that it was a useful asset for COVID-19-related activities, and 88% would use APHVC again. For non-COVID-19-related activities, 24% of respondents agreed there was a strong need for APHVC services. Many respondents (43%) indicated they somewhat agree that APHVC may support LHDs. Most respondents (71%) that used APHVC indicated that APHVC added value and filled gaps in work competencies lacking within their LHD.

Respondents who used APHVC (36%) reported being satisfied with services received; qualitative findings confirmed these responses. Data analysis, data visualization, infographics, and signage were identified as the most valuable services they used and should be kept in future versions of the program.

Respondents who did not use APHVC (60%) reported they tried to use APHVC but had a bad experience or faced various barriers; 44% noted they did not have time or resources to train and supervise volunteers, over 50% said they did not know why their LHD did not use APHVC, and 42% did not know how to engage and did not have enough information to use APHVC. Of respondents who did not use APHVC, 20% had concerns about quality of project/deliverables, 22% had concerns about students' readiness, 46% were concerned about the staff oversight requirements, and 40% reported having no concerns. Additionally, respondents reported that assistance with data analysis (69%), social media (66%), website content (60%), and grant writing (57%) would continue to be beneficial in the future; these responses were confirmed by the qualitative findings.

### Qualitative findings

Eleven survey respondents (14%) participated in a focus group or interview. Findings are presented according to CFIR constructs and separated by APHVC use and non-use (Table 2).

**Table 2:** Examples of Key Qualitative Themes from Local Health Department Respondents (*n* = 11) from 2020 to 2021 by the Consolidated Framework for Implementation Science Construct.

**CFIR Domains: Constructs**	**Respondents: Collaborated with Academic Public Health Volunteer Corp (APHVC)**	**Respondents: Did not collaborate with Academic Public Health Volunteer Corp (APHVC)**
*Intervention characteristics: Relative advantage*	*(+)* APHVC brought outside perspectives and suggestions to the LHD that pushed forward efforts, enabled LHDs think outside the box, and provided services the LHD would otherwise not have	(–) Many LHDs had previous or existing volunteer bases, and access to other resources they could use throughout the pandemic, which was easier than reaching out to new organizations
*Intervention characteristics: Complexity*	*(–)* It was time consuming to train and explain internal processes to volunteers (sometimes easier to do the work themselves) and communicating with volunteers was a challenge often do to unclear expectations and volunteer overcommitment	(–) Managing, training, and coordinating volunteer work required a lot of time and effort. There was concern volunteers would need “handholding
*Outer setting: Patient needs and resources*	*(+/–) LHD*s need mechanisms to gather and analyze data, and often look at data from external sources that indicate needs and health impact of the social determinants to be able to address inequities and health concern in communities	(+) There is a strong need for community needs assessments, share services, and health messaging
*Outer setting: Cosmopolitanism*	*(+)* LHDs feel that people do not understand the role of the LHD, or what they actually do unless they live it. This may cause networking issues and build initial relationships	(–) Individuals who are already part of the community and are trusted (i.e., school nurses) are impactful because of the trust and comfort established
*Inner setting: Tension for change*	*(+)* There is urge for change across LHDs; for growth and sustainability, LHDs require increased staffing support and access to dynamic services. Continuing to discuss needs with the LHD will inform future interventions and resources, and bridge gaps in awareness of what LHD's do	(+/–) The general public does not generally know what local public health departments are responsible for, highlighting the need for increased for transparency and visibility
*Inner setting: Available resources*	*(+)* The lack of available resources is a big reason to leverage APHVC. Lack of funding, lack of time, and lack of ability to train staff. APHVC is a mechanism to build capacity	*(+)* Lack of funding is a root cause for other issues such as understaffing, inability to build capacity, and lack of resources/services
*Characteristics of the individual: Knowledge & beliefs about the intervention*	(–) LHD staff were impacted by lag time on deliverables and lack of communication from volunteers	(+) LHDs hold belief that APHCV can be helpful in enhancing the public health workforce pipeline and are willing to facilitate building relationships
*Characteristics of the individual: Individual identification with organization*	*(+/–)* Individuals in LHDs were overwhelmed and needed additional support prior to the pandemic; the pandemic exacerbated this	*(+/–)* Some LHDs have health equity and community health as core personal values, but integrating them into their LHD work is challenging because of lack of bandwidth and/or support for work beyond regulatory public health work
*Process: Engaging*	*(+)* Engaging students who are excited, interested, and understand what needs to get done is crucial for building good relationships with the LHD's and are the most useful for LHDs	(+/–) LHDs value similar skills of employees as they do volunteers such as communication, critical thinking, independence, writing, epidemiology, etc. Engaging students with these skills would be beneficial
*Process: Planning*	*(–)* Clear expectations of the volunteer at the LHD, and proper transition planning when a volunteer stopped working for and LHD would improve LHD experiences	(+) Having a set/known time commitment (e.g., number of hours a week, total time available, etc.) ahead of time is important for understanding what the volunteer will be capable of executing

#### Intervention characteristics

The intervention characteristics domain identifies key attributes of the intervention that assists in identifying barriers and facilitators that impact the implementation success of the intervention including cost, complexity, and evidence strength.

##### APHVC use

Respondents recognized that volunteers provided high-quality deliverables, brought new perspectives, and encouraged LHDs to think more broadly about their programs. However, they noted that training and supervising volunteers often required more effort and work than if the LHD staff had completed certain projects or work themselves. Challenges included: time required to match, train and oversee a volunteer not familiar with the community; lag times for deliverables; gaps in communication; and, lack of a plan to address problems.

##### APHVC non-use

Those who did not collaborate with APHVC commented on their lack of knowledge of services offered or how to access the program. Many of them utilized pre-existing contract and volunteer services that were easier to access and offered skills beyond traditional public health. For direct community work, respondents indicated they needed people from within the community whom they already knew and trusted. Importantly, they noted having a minimum and standard time commitment from students, and a volunteer liaison would be helpful in training and supervising volunteers.

#### Outer setting

The outer setting refers to the environmental, social, political, and economic context outside of an implementation organization's control and plays a dynamic role with the inner setting. This domain addresses external factors such as policies and incentives, needs and resources, and competitive pressure to implement an intervention.

##### APHVC use

LHDs require data, specifically on health and social inequities within the population, to respond to community needs. During COVID-19, LHDs had access to some state and community-level data, but was not prepared for the rapid COVID-19 response and the need for additional staff and resources (e.g., community response services, mental health, wellness programs). Along with inconsistent COVID-19 communication and frequent changes in mandates, respondents reported that their health departments struggled to determine the best course of action for resolving these issues or addressing community needs.

##### APHVC non-use

Non-use LHDs lacked both awareness of community needs and capacity to conduct a rapid community needs assessment. Their representatives who responded to the survey believed working with trusted individuals within their community, as opposed to external APHVC volunteers, would enhance their COVID-19 response. Moreover, while grant funding would have been beneficial for them to acquire, they indicated it was difficult to seek APHVC assistance due to the rigid requirements for the process and that the service was shared across different jurisdictions.

#### Inner setting

The inner setting domain addresses organizational factors (e.g., available resources, networks and communication, culture, readiness for implementation) that respondents perceive directly impact the implementation.

##### APHVC use

Respondents reported that their LHDs lacked staff and recognized that APHVC volunteers could temporarily fill gaps and provide services that they could not do. Yet, some staff indicated they were unable to grow and develop new services as desired, and lacked staff to launch them. High staff turnover also impacted their capacity to train and supervise short-term volunteers. They were not able to prioritize volunteers over the needs of long-term staff.

##### APHVC non-use

Similarly, non-use LHDs reported limited time and resources as a common reason for not using APHVC; respondents indicated they wanted to conduct community assessments and acquire more funding, staff, data, and resources but were not able to. They noted their LHDs would have sought after APHVC support more if there had been greater compatibility between the services needed and APHVC services offered.

#### Characteristics of individuals

The characteristics of individuals domain address the perceptions, knowledge, confidence, and commitment to change of the individuals within the organization as related to an intervention's success.

##### APHVC use

Respondents reported that their LHD staff found APHVC to be an important learning opportunity for volunteers because they had similar valuable experiences in practice-based programs when they were students. Additionally, after using APHVC, many felt they knew how to best utilize volunteers' time and expertise if there were future iterations of the program. However, issues with deliverable lag time, communication, and limited time of volunteers were challenges associated with using APHVC. They disclosed being overwhelmed and burnt out with the numerous barriers to attracting new employees. They indicated that a valuable aspect of APHVC is its potential for recruiting volunteers to the LHD workforce.

##### APHVC non-use

Non-use LHDs discussed limited bandwidth and time to engage with new programs, highlighting needing to improve APHVC workflow to reduce burden. LHDs desire volunteers who work independently and collaboratively, are willing to learn, and have skills in writing and epidemiology.

#### Process

The process domain considers engagement strategies, delivery of the intervention, leadership roles, and feedback. This domain is important for understanding sustainability, program champions, leadership structure, and networks as well as program evaluation and reflection of processes and protocols.

##### APHVC use

Respondents connected student engagement, enthusiasm, and interest to building strong relationships, increasing the likelihood of ongoing program use. Likewise, clear expectations, volunteer skills, commitment, and ownership reduced their LHDs' burden. Inconsistent communication, commitment, and unrealistic expectations were attributed to negative experiences.

##### APHVC non-use

Respondents from non-use LHDs emphasized the need to have resources for health departments across the state, so they could tap into volunteers who have “valuable” skills (e.g., strong communication skills, ability to use social media to share information, epidemiologic skills, etc.) and/or a volunteer liaison (who can help supervise and train volunteers to facilitate stronger engagement of target communities).

## Discussion

The COVID-19 pandemic exacerbated unmet needs across communities and exposed gaps in the public health infrastructure. Few who receive formal public health training are choosing to work in local public health (24) due to limited benefits, non-competitive wages, and few opportunities for development and advancement (25, 26), While there have been investments made to strengthen the public health workforce in the long-term (27), more rapid and innovative solutions are needed to reduce the current health burden faced by communities (4, 28). Academic-practice based partnerships may help address these needs.

This mixed-methods evaluation (MME) of Academic Public Health Volunteer Corps (APHVC) provided detailed insights into an academic-practice based partnership that informed sustainability and replication of the program. LHDs that utilized APHVC indicated that volunteers filled resource gaps, enhanced their capacity to meet community needs, and created high-quality deliverables. Those that did not engage with APHVC cited insufficient time, bandwidth, and staff to train and engage volunteers, highlighting the need to reduce program burdens. Because of the shortage in staffing and available resources across LHDs and with the potential for academic institutions to provide improved support, initiating partnerships like APHVC could represent a real world solution for helping improve and augment public health services during emergencies like COVID-19 and beyond.

Models like APHVC are well-suited to fill staffing gaps, and could potentially enable LHD staff to apply for grant funding to increase resources. Importantly, these partnerships provide high-quality and diverse services compatible with LHDs' needs and act as a mechanism to build capacity in the local public health workforce. These MME findings are similar to those for other academic and community partnerships that improved the efficiency of work when staffing is low, that increased the number of individuals working in public health, and that brought academic expertise to local public health (29, 30).

LHDs, however often lacked the staff and time to train, communicate with, and supervise short-term student volunteers. This is consistent with other findings in the literature, which confirmed that staffing and the time required to supervise students can be a significant challenge when organizations do not know if the collaboration will improve outcomes or result in return on investment (31). Comprehensive planning to operationalize volunteers (30) and the ability to mitigate time constraints through additional administrative support may be needed and should be encouraged in future iterations of programs.

There are other strategies and activities that APHVC or similar models could explore in the future to potentially optimize a program. While many LHDs expanded and modified services to respond to the pandemic, they also reduced many core functions such as various screening, prevention, and immunization services; services that could have directly impacted transmission, illness severity, comorbidities, and other health outcomes related to the pandemic. APHVC volunteers could have assisted in managing these pre-existing LHD services that were de-prioritized due to the pandemic. LHDs would not only benefit volunteers in learning about the functionality and impact of LHDs in communities, but also could facilitate provision of developed community services to improve community health and to provide a tertiary benefit of supporting pandemic related health outcomes.

Additionally, APHVC and similar model programs could support LHDs in applying different methods of community engagement as well as strategic communication, including using the latest science, techniques, and ideas, discussed in coursework to support and inform disease prevention guidance for target communities. Many LHDs relied heavily on expectations and federal and state authority to act and engage, often without robust outreach or messaging tailored to target audiences. APHVC volunteers could have brought innovative ideas and deep knowledge and skills in new technologies and engagement. Ultimately, volunteers could serve as an extra resource to support departments in modifying interventions so they better meet community needs, build trust, reinforce collaboration in developing and utilizing various modes of communication, and increase the depth of knowledge and services to inform public health policy and practice.

Despite existing barriers, LHDs and academic institutions can have a reciprocal relationship leading to quality improvements in the provision of public health services (32, 33). The MME findings suggest that LHDs could work with APHVC to enhance the public health workforce pipeline and increase the visibility of local public health while providing students with real-world experience. Public health workforce and infrastructure development are ongoing priorities for national government agencies (34), and the bi-directional relationship between LHDs and academic institutions could and should continue to help build capacity and provide further resources for the public health workforce.

Evaluating this mass mobilization of public health volunteers through an implementation science framework produced tangible recommendations (Table 3) for APHVC's implementing agency and for any institution seeking to support local public health infrastructure. The MME informed changes to the APHVC, which was redeveloped into the Academic Public Health Corps (APHC) in 2021 and has been formalized as an academic-practice base partnership work education program to support public health agencies across the state of Massachusetts. The APHC is currently undergoing a longer-term MME that seeks to build on the work presented herein. This longer-term MME will work to understand if the program is being implemented as intended, measuring impact on its target population, meeting program goals, and much more.

**Table 3:** Recommendations to the implementing agency in 2022 to Improve the Academic Public Health Volunteer Corps.

**Consolidated Framework for Implementation Research (CFIR) domain**	**CFIR construct**	**Barrier or facilitator**	**Recommendation for APHVC**
*Intervention characteristics*	*Relative advantage*	*Facilitator*	Sustain the variety of diverse services so that LHDs of a “menu” of services they can access as needed
	*Complexity*	*Barrier*	Create a more efficient matching volunteer-LHD process to appropriately connect volunteers with certain skills to communities that need those skills
*Outer setting*	*Cosmopolitanism*	*Facilitator*	Previously existing relationships with other resources and communities should be leveraged in conjunction with APHVC for maximum benefit
*Inner setting*	*Available Resources*	*Facilitator*	APHVC should frame themselves as having the ability to fill resource gaps without adding extra burden to the LHD
	*Compatibility*	*Barrier*	Consider an APHC model that has more on-going collaborations or resources available to LHDs (i.e., faculty, health educators, etc.) that maintain continuity as students move in and out each semester
*Characteristics of the individual*	*Knowledge & Beliefs about the Intervention*	*Barrier*	Enhance marketing and outreach about the APHC to improve ease of access to information about and engagement with the APHC
*Process*	*Planning*	*Facilitator*	Have a liaison for LHDs to work with that will streamline communication, relieve the LHDs of certain time-consuming tasks like training and onboarding, and will improve engagement and manage expectations between health departments and students

### Strengths and limitations

To our knowledge, this particular academic-practice based partnership is unique among those found in the literature. APHVC is the first state-wide partnership available to any LHD, to public health students, and alumni from multiple universities, as compared to a specifically collaboration between a single LHD and a single university. To our knowledge, it is the only evaluated academic-practice based partnership developed during COVID-19 that mobilized public health students.

The MME has several limitations. First, the evaluation included students who previously volunteered for APHVC. While this provided greater insights into program operations from students, this nuanced aspect of the evaluation may have also biased the overall objectivity of the evaluation analyses. Second, LHD staff remained overstretched during the MME. Thus, the qualitative component of the evaluation was less robust (i.e., the program evaluation would have benefited from more participation of LHD stakeholders in the focus groups and interviews). Finally, findings and lessons learned may not be generalizable because health department structures and resources vary from state to state during the pandemic, often operating in a variable fashion, as a de-centralized system (such as in this case), as a centralized system, or as some combination of both.

Future research should expand the assessment of stakeholders to better understand their varying perspectives, their needs, and how to best prepare them for working with volunteers, so that APHVC experiences are mutually beneficial. Continual evaluation of model programs like APHVC will yield greater understanding of how they work and enable LHDs to make informed decisions and changes required to improved community health and individual health-related outcomes.

### Public health implications

This MME encapsulates the multi-faceted dynamics of implementing a novel academic-practice based program that can be used to improve program operations on a local or national scale. The repercussions and health and social impacts caused by the COVID-19 pandemic endure, and local public health is more resource-constrained than ever before to address emerging and ongoing threats to the health of communities. Partnerships like APHVC can help strengthen the LHD workforce, mitigate the effects of a health crisis, and build LHD capacity to better meet community needs regardless of emergencies. Such programs are also beneficial to early public health professionals, who can practical skills and may join the public health workforce after graduating from school. With limited examples of these partnerships, working to understand them and improve existing partnerships through program evaluation will be key actions for enhancing public health preparedness and capacity to address public health core functions in the post COVID-19 recovery era.

## Data availability statement

The raw data supporting the conclusions of this article will be made available by the authors, without undue reservation.

## Ethics statement

The studies involving humans were approved by Boston University Institutional Review Board. The studies were conducted in accordance with the local legislation and institutional requirements. Written informed consent for participation was not required from the participants or the participants' legal guardians/next of kin because this study was approved as exempt. Verbal informed consent was obtained and approved.

## Author contributions

SF: Data curation, Formal analysis, Investigation, Writing – original draft, Writing – review & editing. PE: Conceptualization, Data curation, Funding acquisition, Investigation, Methodology, Project administration, Resources, Supervision, Writing – review & editing, Writing – original draft. AC: Data curation, Formal analysis, Writing – review & editing. HA: Data curation, Formal analysis, Project administration, Writing – original draft, Writing – review & editing. AC: Data curation, Formal analysis, Writing – review & editing. ZW: Data curation, Formal analysis, Writing – review & editing. JG: Conceptualization, Data curation, Formal analysis, Funding acquisition, Investigation, Methodology, Project administration, Resources, Supervision, Writing – original draft, Writing – review & editing.
